# SIGLEC1 (CD169): a marker of active neuroinflammation in the brain but not in the blood of multiple sclerosis patients

**DOI:** 10.1038/s41598-021-89786-0

**Published:** 2021-05-13

**Authors:** Lennard Ostendorf, Philipp Dittert, Robert Biesen, Ankelien Duchow, Victoria Stiglbauer, Klemens Ruprecht, Judith Bellmann-Strobl, Dominik Seelow, Werner Stenzel, Raluca A. Niesner, Anja E. Hauser, Friedemann Paul, Helena Radbruch

**Affiliations:** 1 Department of Neuropathology, Charité-Universitätsmedizin Berlin, Corporate Member of Freie Universität Berlin, Humboldt-Universität zu Berlin, Berlin Institute of Health, Berlin, Germany; 2 Rheumatology and Clinical Immunology, Charité-Universitätsmedizin Berlin, Corporate Member of Freie Universität Berlin, Humboldt-Universität zu Berlin, Berlin Institute of Health, Berlin, Germany; 3 Deutsches Rheuma-Forschungszentrum Berlin, A Leibniz Institute, Berlin, Germany; 4 RG Bioinformatics and Translational Genetics, Berlin Institute of Health (BIH), Berlin, Germany; 5 NeuroCure Clinical Research Center, Charité-Universitätsmedizin Berlin, Corporate Member of Freie Universität Berlin, Humboldt-Universität zu Berlin, Berlin Institute of Health, Berlin, Germany; 6 Department of Neurology, Charité-Universitätsmedizin Berlin, Corporate Member of Freie Universität Berlin, Humboldt-Universität zu Berlin, Berlin Institute of Health, Berlin, Germany; 7 Clinical and Experimental Multiple Sclerosis Research Center, Charité-Universitätsmedizin Berlin, Corporate Member of Freie Universität Berlin, Humboldt-Universität zu Berlin, Berlin Institute of Health, Berlin, Germany; 8 Experimental and Clinical Research Center, Max Delbrück Center for Molecular Medicine & Charité–Universitätsmedizin Berlin, Corporate Member of Freie Universität Berlin, Humboldt – Universität zu Berlin, Berlin Institute of Health, Berlin, Germany; 9 Institute of Medical Genetics and Human Genetics, Charité-Universitätsmedizin Berlin, Corporate Member of Freie Universität Berlin, Humboldt-Universität zu Berlin, Berlin Institute of Health, Berlin, Germany; 10 Fachbereich Veterinärmedizin, Institute of Veterinary Physiology, Freie Universität Berlin, Berlin, Germany

**Keywords:** Neuroimmunology, Neurological disorders

## Abstract

We aimed to evaluate SIGLEC1 (CD169) as a biomarker in multiple sclerosis (MS) and Neuromyelitis optica spectrum disorder (NMOSD) and to evaluate the presence of SIGLEC1^+^ myeloid cells in demyelinating diseases. We performed flow cytometry-based measurements of SIGLEC1 expression on monocytes in 86 MS patients, 41 NMOSD patients and 31 healthy controls. Additionally, we histologically evaluated the presence of SIGLEC1^+^ myeloid cells in acute and chronic MS brain lesions as well as other neurological diseases. We found elevated SIGLEC1 expression in 16/86 (18.6%) MS patients and 4/41 (9.8%) NMOSD patients. Almost all MS patients with high SIGLEC1 levels received exogenous interferon beta as an immunomodulatory treatment and only a small fraction of MS patients without interferon treatment had increased SIGLEC1 expression. In our cohort, SIGLEC1 expression on monocytes was—apart from those patients receiving interferon treatment—not significantly increased in patients with MS and NMOSD, nor were levels associated with more severe disease. SIGLEC1^+^ myeloid cells were abundantly present in active MS lesions as well as in a range of acute infectious and malignant diseases of the central nervous system, but not chronic MS lesions. The presence of SIGLEC1^+^ myeloid cells in brain lesions could be used to investigate the activity in an inflammatory CNS lesion.

## Introduction

Multiple sclerosis (MS) and Neuromyelitis optica spectrum disorder (NMOSD) are both chronic, demyelinating inflammatory diseases of the central nervous system that are a significant cause of disability in the young. While certain aspects of the pathophysiology of both diseases have been uncovered, many aspects towards a complete understanding remain to be elucidated^1^.

SIGLEC1 (Sialic acid-binding immunoglobulin-type lectins-1, CD169) is a sialic acid binding cell-surface protein, exclusively expressed on monocytes and macrophages^2^. Its expression is upregulated upon contact with type I interferons and to a lesser degree, other activatory stimuli such as LPS^3,4^. As such, SIGLEC1 expression has been used as a surrogate marker for type I interferon activity in autoimmune diseases like Systemic lupus erythematosus^5^ or primary Sjögren syndrome^6^ as well as interferonopathies^7^ and viral infections^8,9^.

In the healthy brain, SIGLEC1 is only expressed by some perivascular and choroid plexus macrophages, but not by microglia^10^. In mice, mechanical insult to the brain led to an accumulation of SIGLEC1^+^ myeloid cells in the damaged area^10^. The cause of this accumulation, however, is unclear—either the contact of serum components or inflammatory signals with resident myeloid cells induces SIGLEC1 expression or blood-derived SIGLEC1^+^ myeloid cells infiltrate the CNS.

The role of type I interferons and SIGLEC1 in the pathophysiology of MS and NMOSD is not yet clear. In relapsing–remitting MS (RRMS), type I interferons are used as an immunomodulatory treatment that reduces the rate of relapses^11^. It does, however, not protect against clinical progression in the progressive forms of MS (primary progressive MS (PPMS) and secondary progressive MS (SPMS))^11^. In a recent report, SIGLEC1 positive myeloid cells were found within MS lesions and specific ablation of SIGLEC1 expressing cells in the experimental autoimmune encephalitis (EAE) model of MS led to an amelioration of disease^12^. Additionally, plasmacytoid dendritic cells, the main type I interferon-producing cells, were found in increased frequencies in the cerebrospinal fluid of MS patients during a flare^13^. Two studies found SIGLEC1 expression to be increased on blood monocytes of MS patients, especially those with a progressive type of MS^12,14^. Taken together, there is conflicting evidence on the role of type I interferons and the interferon-induced expression of SIGLEC1 in MS as being protective or pathogenic. This is likely due to the heterogeneity of patients and disease stages and potentially also the confounding effect of interferon therapy.

In NMOSD, there is some evidence that type I interferons play a role in its pathogenesis: many patients with NMOSD have an overlap with additional, type I interferon-dependent diseases, such as SLE^15^ and a recent report describes a series of patients with increased levels of endogenous or exogenous interferon α who went on to develop a seropositive NMOSD^16^. Interferon treatment does not prevent NMOSD relapses^17^ and anecdotally even increases the disease activity^18^. Transcriptomic profiling of blood from NMOSD patients identified a “interferon high” signature in 16 of 38 (42.1%) of NMOSD patients^19^. This signature included the transcript for SIGLEC1 and was associated with higher disease severity. Additionally, we recently described the presence of low-density granulocytes in MS and NMOSD patients^20^, a subset of granulocytes that produce high levels of type I interferons in SLE^21^. Thus, we hypothesized that type I interferons could also play a role in the pathogenesis of NMOSD.

In this work we investigated expression of SIGLEC1 on monocytes of patients with MS and NMOSD and correlated the expression with clinical parameters. In addition, we analysed human brain tissue sections of active, inflammatory and chronic MS lesions as well as other neurological and systemic diseases.

## Results

### SIGLEC1 expression is increased in a subset of patients with multiple sclerosis

**Figure 1:**
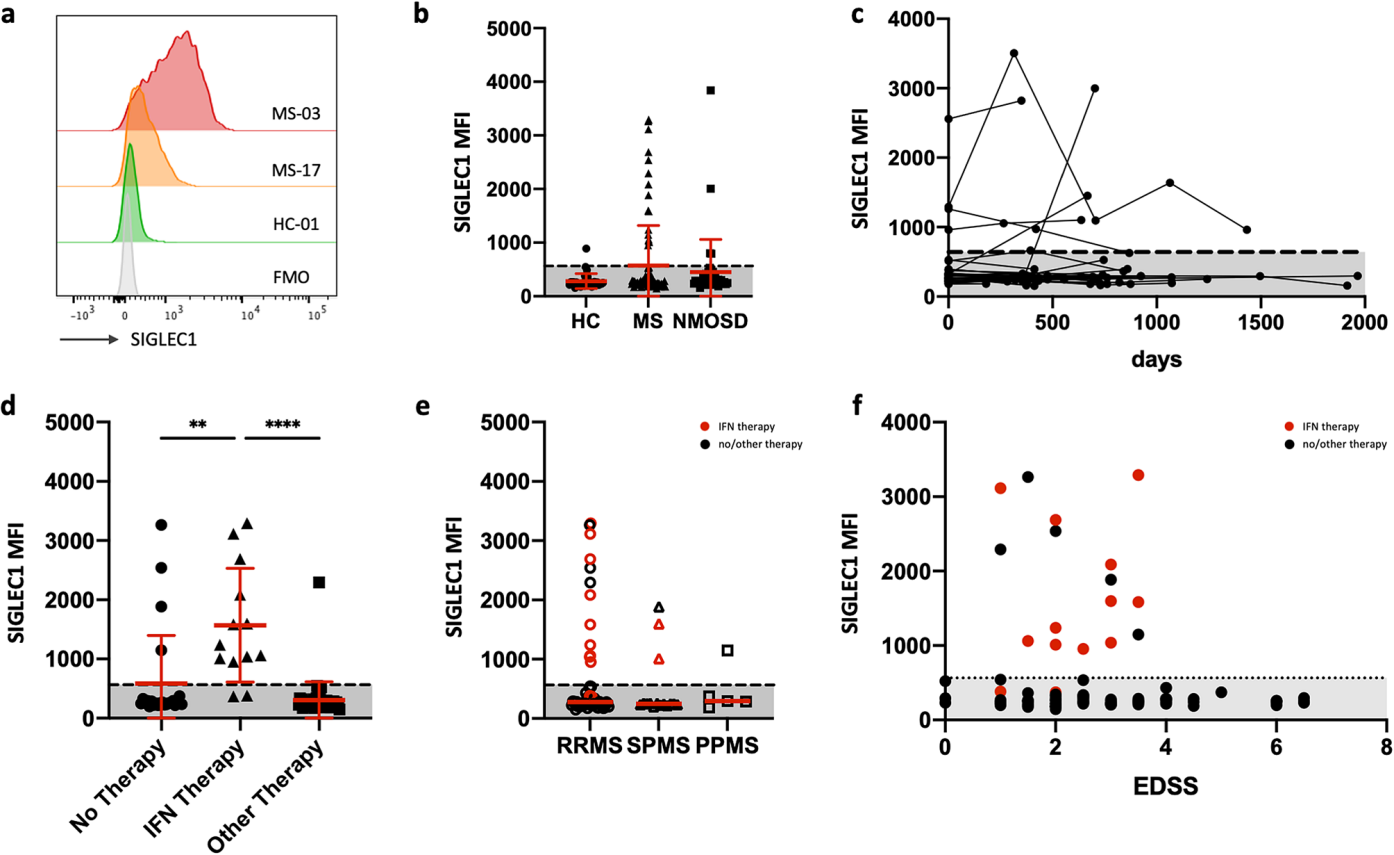
(**a**) Representative histograms of SIGLEC1 fluorescence on CD14^high^ monocytes, FMO: fluorescence minus one control. (**b**) Cross-sectional analysis of SIGLEC1 expression on CD14^high^ monocytes of 31 healthy controls, 86 MS patients and 41 NMOSD patients. If multiple samples of the same individual were analysed, the mean of the measurements is displayed. (**c**) Longitudinal analysis of SIGLEC1 expression of 52 individuals with up to five measurements. (**d**) Comparison of SIGLEC1 expression in MS patients, receiving no immunomodulatory treatment (n = 24), interferon beta (n = 13) or a non-interferon treatment (n = 49). Treatment with interferon with significantly associated with higher levels of SIGLEC1 expression: Kruskal–Wallis test with Dunn’s correction, **p < 0.01, ****p < 0.0001. (**e**) Comparison of SIGLEC1 expression in patients with RRMS (n = 63), SPMS (n = 15) and PPMS (n = 5). Red symbols indicate interferon treatment. (**f**) Scatter plot of the EDSS disability score against SIGLEC1 expression, no significant correlation was detected.

We investigated the SIGLEC1 expression on CD14^+^ monocytes in the peripheral blood of MS and NMOSD patients as well as in healthy controls. In most of the samples, monocyte SIGLEC1 expression was barely above the negative (fluorescence minus one) control (Fig. 1a,b). We defined a physiologic range of SIGLEC1 expression as two standard deviations from the mean SIGLEC1 expression in healthy controls (normal MFI range 0–564). Accordingly, 16/86 (18.6%) MS patients, 4/41 (9.8%) NMOSD patients and 1/31 (3.2%) healthy controls had increased SIGLEC1 levels. Almost all individuals with available longitudinal samples remained in their respective SIGLEC1 high or low category over a follow-up period of up to 1965 days. (Fig. 1c).

### Treatment with type 1 interferon explains almost all increased

Next, we aimed to identify parameters that were associated with increased SIGLEC1 in MS patients. One obvious explanation would be exogenous type 1 interferon as treatment. 11/16 MS patients (68.6%) with increased SIGLEC1 expression levels received interferon beta treatment at the time of measurement (Fig. 1d). While a previous report found increased SIGLEC1 levels to be more prevalent in MS patients with a progressive form of MS, we found only 2/20 patients primary or secondary progressive MS with increased SIGLEC1 levels that were not explained by interferon treatment (Fig. 1e). SIGLEC1 expression on monocytes did not correlate with the Expanded Disability Status Scale (EDSS) (Fig. 1f), nor had it a temporal association with relapses. In the NMOSD patients, one of the four patients with increased SIGLEC1 expression had the additional diagnosis of a mixed connective tissue disease (MCTD), a disease that is also associated with increased type 1 interferon activity^22^.

### SIGLEC1 expression on brain-infiltrating myeloid cells

**Figure 2:**
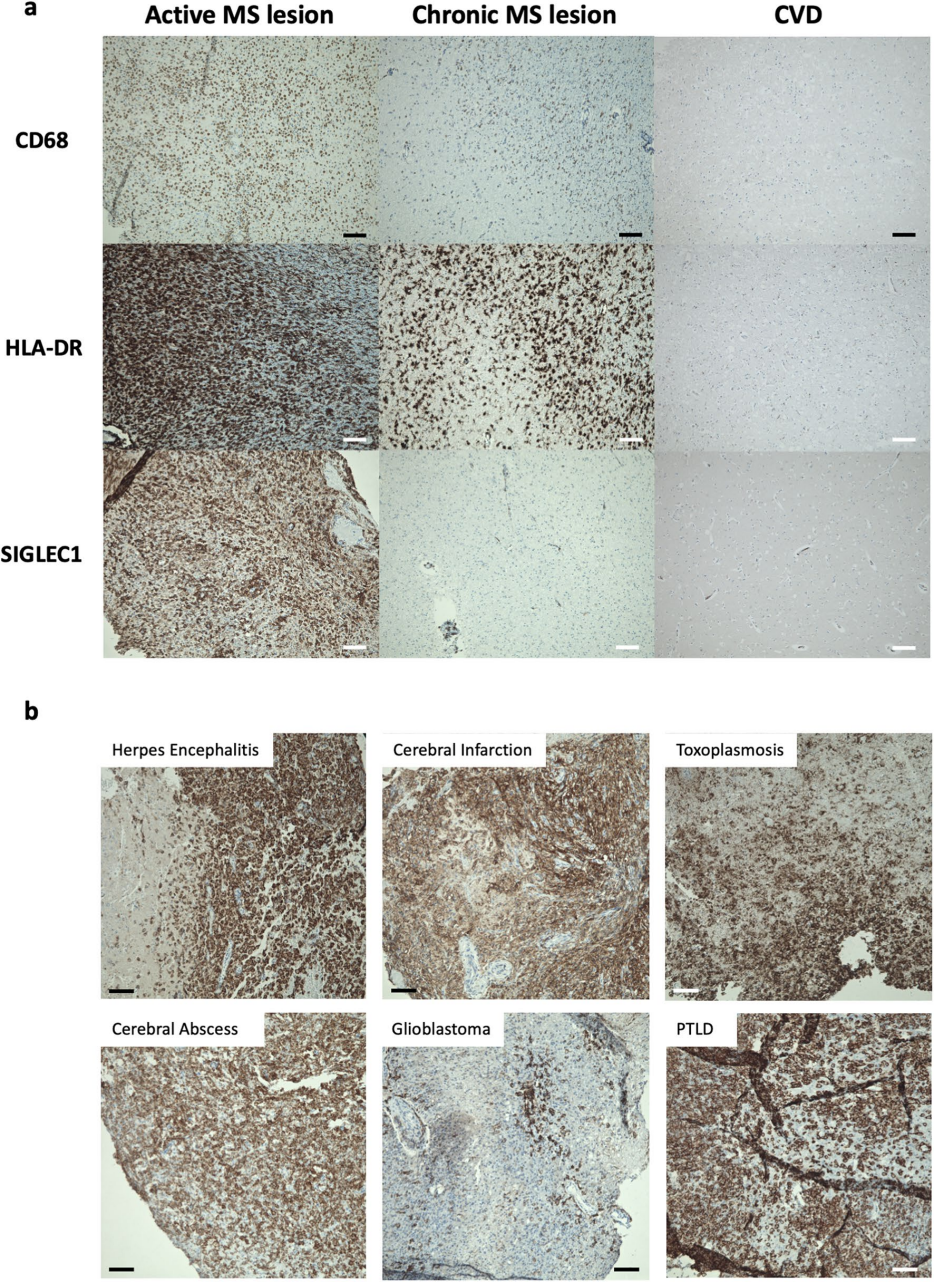
(**a**) Representative immunohistochemical stainings of CD68, HLA-DR and SIGLEC1 (CD169) in brain tissue from a patient with active inflammatory multiple sclerosis lesion, a chronic lesion from a patient with secondary-progressive multiple sclerosis (SPMS) and a control patient who died of cardiovascular disease (CVD). Both RRMS and SPMS show infiltrates with CD68^+^ and HLA-DR^+^ myeloid cells, but only in the active lesion, these express SIGLEC1. Nuclei are stained blue and the respective marker antigens in brown. The scale bar indicates 100 µm. These results are representative of 4 RRMS, 5 SPMS and 7 control samples that were examined. (**b**) Immunohistochemical stainings of SIGLEC1 in brain tissue from patients with Herpes simplex viral encephalitis (n = 2), cerebral infarction with inflammatory changes (n = 2), cerebral toxoplasmosis (n = 1), cerebral abscess (n = 2), glioblastoma (n = 2) and post-transplant lymphoproliferative disease (PTLD, n = 1). Infiltrates of SIGLEC1^+^ myeloid cells were observed in all samples.

To investigate the presence and specificity of SIGLEC1+ myeloid cells in inflammatory MS lesion, we analysed brain tissue sections from 4 patients with MS lesions that had clinical, radiological and histological signs of activity (gadolinium uptake on MRI imaging, presence of myelin-laden macrophages), 5 patients with secondary-progressive MS (SPMS) who died of acute non-neurologic causes and with histologically classified chronic lesions and 6 patients who died of cardiovascular or multi-organ failure. In all control samples, SIGLEC1 positivity was limited to cells in the leptomeninges as well as perivascular macrophages, as previously reported^10^. In all four samples from patients with active MS lesions as well as in one patient with the Marburg variant of MS who died of the disease 14 days after onset, we found a dense infiltrate of CD68+HLA-DR+ myeloid cells that stained predominantly positive for SIGLEC1 (Fig. 2a). In contrast, in five samples from patients with secondary-progressive MS (SPMS) with histologically classified chronic lesions, we found CD68+HLA-DR+ infiltrates of varying density, but almost no SIGLEC1 expression (Fig. 2a). This indicates that SIGLEC1 expression on myeloid cells could be used to distinguish active inflammatory lesions from chronic lesions.

To corroborate this hypothesis, we studied the SIGLEC1 expression in the brain tissue of 8 patients with other inflammatory neurological diseases. SIGLEC1+ myeloid infiltrates were present in patients with glioblastoma (n = 2), herpes simplex encephalitis (n = 2) and cerebral infarction (n = 2), as well as toxoplasmosis (n = 1), cerebral abscess (n = 2) and post-transplant lymphoproliferative disorders (n = 1, PTLD, Fig. 2b). No increase in SIGLEC1+ cells was noted in the brain of a patient who died of amyotrophic lateral sclerosis (ALS).

In summary, SIGLEC1+ is expressed on brain-infiltrating myeloid cells in a broad range of active inflammatory lesions, but not in chronic MS lesions.

## Discussion

Here, we report that, after accounting for interferon treatment, patients with multiple sclerosis (MS) and neuromyelitis optica spectrum disorder (NMOSD) did not have increased expression of SIGLEC1 on monocytes in the peripheral blood.

We identified SIGLEC1+ on CD68+ HLA-DR+ myeloid cells in active inflammatory MS lesions and a range of other inflammatory, infectious or malignant brain lesions. SIGLEC1+ expression was low on myeloid cells in chronic MS lesions of SPMS patients, indicating that SIGLEC1+ myeloid cells could serve as a marker of inflammatory activity. These findings are in line with a previous report that found SIGLEC1+ MHC-II+ myeloid cells to be present in mice after retinal transplantation, but not in the healthy retina or retinal degeneration^23^. Future studies will be needed to investigate, whether the increase in SIGLEC1+ cells in inflammatory brain lesions is due to the infiltration of SIGLEC1+ cells from the peripheral blood or an upregulation of SIGLEC1 in tissue-resident microglia, as well as define the inflammatory signals leading to such an upregulation. We propose that SIGLEC1+ can serve as a marker to differentiate active from inactive MS lesions, which will require additional validation for use in routine histopathological diagnostics.

Our results on SIGLEC1 expression on blood monocytes are in contrast to two previous reports which identified increased levels of SIGLEC1 in cohorts of 44 MS patients^12,14^. The study by Malhotra et al.^14^ focussed on untreated MS patients and identified modest increases in SIGLEC1 expression in MS patients (approximately twofold increase of MFI between MS patients and HC) and especially those with progressive disease. A second study by Bogie et al.^12^ again identified small (approximately twofold) increases in SIGLEC1 expression on monocytes in 57 MS patients and found that these increases were independent of progressive disease and also of interferon treatment, an observation that is at odds with our own findings and biological plausibility.

This disparity might be due to different reasons: it could be that the authors mostly observed small differences between individuals that would have all been classified as “SIGLEC1 low expressors” in our study, as the approximately twofold-differences they describe are significantly smaller compared to the five–tenfold increases in SIGLEC1 MFI in “SIGLEC1 high expressors” in our study. Different studies investigated the SIGLEC1 expression using different antibody clones which could also explain some of the differences; in this study, we used the same clone as previous studies that investigated SIGLEC1 in rheumatologic diseases^5,6^.

Additionally, our data are at odds with a previous report that found a type 1 interferon signature in NMOSD patients that was conducted with patients from the same patient cohort^19^. While 42% of NMOSD cohorts in the study by Agasing et al. showed an “high type 1 interferon” signature, we found < 10% of NMOSD patients had increased type 1 interferon activity as measured by SIGLEC1 expression; a result that is neither significantly different from healthy controls, nor likely of clinical consequence. As the results of whole blood bulk transcriptomic data are often subject to unforeseen external influences and changes in the cellular composition of the blood, they should usually be validated by protein level data.

Even though our data are not in agreement with multiple, previously published results, the validity of our assay is supported by the internal plausibility control of interferon treated MS patients. However, one significant weakness of our own study is the relative clinical quiescence of the patients enrolled; most did not have a relapse within the last six months before the measurement and most did not have a change in EDSS in longitudinal sampling. Additionally, most patients were under immunosuppressive treatment. Future studies in MS and NMOSD patients during a clinical relapse could thus still discover the presence of SIGLEC1 expressing monocytes in the peripheral blood.

In summary, our data indicate that type I interferons or SIGLEC1 expressing cells in the peripheral blood do not play a major role in the pathogenesis of most patients with stable NMOSD or MS, however SIGLEC1^+^ myeloid cells in the brain are present in active inflammatory MS lesions as well as in other inflammatory neurological diseases of the CNS.

## Methods

### Cohort

We analysed frozen peripheral blood mononuclear cells (PBMCs) from a total of 86 MS patients, 41 NMOSD patients and 31 healthy controls (HCs) that were included in observational cohort studies for MS and NMOSD at the NeuroCure Clinical ResearchCenter, Charité—Universitätsmedizin Berlin. PBMCs from all patients with sufficient available material were included in the study with no prior selection according to disease activity, treatment, age or sex. All MS patients fulfilled the 2017 revised McDonald criteria^24^, while the NMOSD patients fulfilled the 2015 Wingerchuck international consensus diagnostic criteria^25^. Further patient characteristics are provided in Table 1.

**Table 1:** Epidemiological data.

	Healthy controls	MS patients	NMOSD patients
Number of individuals	31	86	41
Age (median, IQR)	31 (27–34)	45 (34–52)	57 (45–64)
Sex (n female)	15 (48.4%)	53 (61.6%)	37 (90.2%)
Subtype (n)	–	RRMS: 63 (73.3%) SPMS: 15 (17.4%) PPMS: 5 (5.9%)	AQP4+: 23 (56.1%) MOG+: 7 (17.1%) Seronegative: 3 (7.3%) Unknown: 8 (19.5%)
Additional immune-mediated diseases (n)	Autoimmune thyroiditis (2), asthma bronchiale (1)	Autoimmune thyroiditis (10), asthma bronchiale (2), psoriasis (1), idiopathic myocarditis (1), celiac disease (1)	Autoimmune thyroiditis (3), myasthenia gravis (1), Sjögren syndrome (1), MCTD (1)
Immunosuppressive medication (n)	–	None: 24 (27.9%) Dimethyl fumarate: 15 (17.4%), glatiramer acetate: 14 (16.3%), interferon beta: 13 (15.1%), fingolimod: 7 (8.1%), teriflunomide: 5 (5.8%), methylprednisolone: 2 (2.3%), daclizumab: 2 (2.3%), rituximab: 1 (1.2%), ocrelizumab: 1 (1.2%), natalizumab 1 (1.2%)	Rituximab: 19 (46.3%) Azathioprine: 9 (22.0%) None: 6 (14.6%) Prednisolone: 3 (7.3%) Mycophenolate: 2 (4.9%) Glatiramer acetate: 1 (2.4%) Unknown: 1 (2.4%)

*IQR* interquartile range, *RRMS* relapse-remitting multiple sclerosis, *SPMS* secondary progressive multiple sclerosis, *PPMS* primary progressive multiple sclerosis, *AQP4*+ aquaporin-4 antibody positive, *MOG*+ myelin oligodendrocyte glycoprotein antibody positive, *MCTD* mixed connective tissue disease).

Longitudinal samples were available for 28/86 MS patients, 12/41 NMOSD patients and 12/31 healthy controls. Follow-up periods were up to 1965 days and included 2 to 5 time points.

### PBMC isolation and flow cytometry

Peripheral blood mononuclear cells (PBMCs) were isolated from EDTA anticoagulated blood. Blood and phosphate-buffered saline supplemented with 0.5% bovine serum albumin (PBS/BSA) were mixed at a 1:1 ratio and 35 ml were layered onto 15 ml Ficoll-Paque PLUS gradient (GE Healthcare). PBMCs were isolated according to the manufacturer’s protocol and stored in liquid nitrogen until the analysis.

For analysis batches of 20–35 samples were thawed and washed in RPMI 1640 cell medium (Gibco). In each batch, a mix of samples from healthy controls and MS/NMOSD patients were included. Cells were stained with antibodies against CD14 (DRFZ, clone TM1), CD169 (SIGLEC1, clone 7–239, BioLegend Cat# 346004, RRID:AB_2189029) and a dead cell stain (eBioscience Fixable Viability Dye eFluor 780). For each batch, a fluorescence minus one (FMO) control for SIGLEC1 was measured and FMO measurements remained stable over multiple batches. The samples were acquired on a FACSCanto cytometer (BD Biosciences) and all cytometry experiments were performed according to published standards^26^. Using the FlowJo software (Version 10.4.1 for Mac, FlowJo LLC), we identified monocytes and excluded doublets based on scatter parameters. We then gated on CD14^high^, living cells and analysed the median fluorescence intensity (MFI) for SIGLEC1 (Fig. 1a).

### Histology of human brain tissue

We investigated archived cryo- and formalin preserved biopsy and autopsy tissue from patients who had been diagnosed in the Department of Neuropathology, Charité—Universitätsmedizin Berlin with inflammatory demyelination of the central nervous system (CNS) consistent with multiple sclerosis or other inflammatory, malignant or infectious diseases of the CNS (other neurological disease, OND). Tissue that was categorized as active MS lesions stemmed from patients with clinical and MRI evidence of active disease with gadolinium contrast agent uptake and/or perifocal edema in T2-weighted sequences. These tissue samples were all derived from diagnostic biopsies that were performed to diagnose an unclear tumefactive lesion that was diagnosed as inflammatory demyelinating lesion. After follow-up, these patients were then clinically diagnosed with MS. Histologically these lesions showed all active demyelination with myelin laden macrophages. In contrast, the patients with lesions categorized as chronic MS lesions were patients diagnosed with SPMS that had died of a non-neurologic cause and where only chronically active or inactive lesions with lack of myelin-laden macrophages were analysed^27^. Patients who died of cardiovascular cases or multi-organ failure served as controls. All patients with multiple sclerosis fulfilled clinical diagnosis criteria according to the 2017 revised McDonald criteria^24^ and none of the patients were treated with interferon.

### Immunohistology of human brain tissue

All stains were performed on 8 μm cryomicrotome sections or 4-μm-thick FFPE tissue sections according to standard procedures. Stains included Hematoxylin and eosin (H&E), CD68 (EBM11, Dako #M0718, 1:100), HLA-DR (CR3/43; Dako #M0775, 1:200) and SIGLEC1 (HSn7D2, Novus Biologicals, 1:200). Immunohistochemical stainings were performed on a Benchmark XT autostainer (Ventana Medical Systems, Tuscon, AZ, USA) with standard antigen retrieval methods for the FFPE sections (CC1 buffer, pH8.0, Ventana Medical Systems, Tuscon, AZ, USA). The presence of SIGLEC1 perivascular and leptomeningeal staining served as an internal quality control. Microscopy was performed on a BZ-9000 BioRevo microscope (Keyence, Neu-Isenburg, Germany). Positively stained cells were defined by presence of a nucleus and cytoplasmic staining.

### Data analysis

Statistical analyses were performed using Python 3.7.6 with the Numpy (v1.19.0)^28^ and Pandas (v1.1.0)^29^ packages as well as GraphPad Prism (v8.4.3. for Mac). A normal range for SIGLEC1 expression in FACS expression was calculated as two standard deviations from the mean SIGLEC1 expression in healthy controls. When multiple measurements of the same individual were available, the mean of the measurements was calculated and used for comparative analysis. The Chi-squared test or Kruskal–Wallis test with Dunn’s correction was used to compare between the different groups for binary and continuous variables respectively.

### Ethics approval and consent to participate

The analysis of blood was approved by the ethics committee of the Charité—Universitätsmedizin Berlin (MS: EA1/163/12; NMOSD: EA1/041/14) and informed consent was obtained from all participants in the study. The study of brain histology was approved approved by the ethics committee of the Charité—Universitätsmedizin (EA1/078/16). This study was performed in accordance with all relevant guidelines and regulations, such as the Declaration of Helsinki and the Good Clinical Practice guidelines of the International Council for Harmonization.

## Data Availability

All datasets used and analysed during the current study are available from the corresponding authors on request.
